# Discrepant Phenotyping of Monocytes Based on CX3CR1 and CCR2 Using Fluorescent Reporters and Antibodies

**DOI:** 10.3390/cells13100819

**Published:** 2024-05-10

**Authors:** Katrin Sommer, Hilal Garibagaoglu, Eva-Maria Paap, Maximilian Wiendl, Tanja M. Müller, Imke Atreya, Gerhard Krönke, Markus F. Neurath, Sebastian Zundler

**Affiliations:** 1 Department of Medicine 1, University Hospital Erlangen, Friedrich-Alexander-Universität Erlangen-Nürnberg, 91054 Erlangen, Germany; katrin.sommer@uk-erlangen.de (K.S.); eva-maria.paap@uk-erlangen.de (E.-M.P.); tanja.mueller@uk-erlangen.de (T.M.M.); imke.atreya@uk-erlangen.de (I.A.); gerhard.kroenke@charite.de (G.K.); markus.neurath@uk-erlangen.de (M.F.N.); 2 Department of Medicine 3, University Hospital Erlangen, Friedrich-Alexander-Universität Erlangen-Nürnberg, 91054 Erlangen, Germany; hilal.garibagaoglu@uk-erlangen.de; 3 Deutsches Zentrum Immuntherapie (DZI), University Hospital Erlangen, 91054 Erlangen, Germany; 4 Medical Department of Rheumatology and Clinical Immunology, Charité—Universitätsmedizin Berlin, 10117 Berlin, Germany

**Keywords:** classical monocytes, non-classical monocytes, C-X3-C motif chemokine receptor 1 (CX3CR1), chemokine (C-C motif) receptor 2 (CCR2), lymphocyte antigen 6C (Ly6C)

## Abstract

Monocytes, as well as downstream macrophages and dendritic cells, are essential players in the immune system, fulfilling key roles in homeostasis as well as in inflammatory conditions. Conventionally, driven by studies on reporter models, mouse monocytes are categorized into a classical and a non-classical subset based on their inversely correlated surface expression of Ly6C/CCR2 and CX3CR1. Here, we aimed to challenge this concept by antibody staining and reporter mouse models. Therefore, we took advantage of *Cx3cr1*^GFP^ and *Ccr2*^RFP^ reporter mice, in which the respective gene was replaced by a fluorescent reporter protein gene. We analyzed the expression of CX3CR1 and CCR2 by flow cytometry using several validated fluorochrome-coupled antibodies and compared them with the reporter gene signal in these reporter mouse strains. Although we were able to validate the specificity of the fluorochrome-coupled flow cytometry antibodies, mouse Ly6C^high^ classical and Ly6C^low^ non-classical monocytes showed no differences in CX3CR1 expression levels in the peripheral blood and spleen when stained with these antibodies. On the contrary, in *Cx3cr1*^GFP^ reporter mice, we were able to reproduce the inverse correlation of the CX3CR1 reporter gene signal and Ly6C surface expression. Furthermore, differential CCR2 surface expression correlating with the expression of Ly6C was observed by antibody staining, but not in *Ccr2*^RFP^ reporter mice. In conclusion, our data suggest that phenotyping strategies for mouse monocyte subsets should be carefully selected. In accordance with the literature, the suitability of CX3CR1 antibody staining is limited, whereas for CCR2, caution should be applied when using reporter mice.

## 1. Introduction

Monocytes are widely conserved cells of the myeloid lineage. In the peripheral blood of mice, they account for up to 4% of all leucocytes [1,2] and develop from hematopoietic stem cells (HSCs) in the bone marrow via the common myeloid progenitor [3]. They are recruited from the bloodstream to the surrounding tissue, where they can differentiate into macrophages or dendritic cells (DCs) dependent on the tissue environment [4].

Monocytes are known to play a central role in both innate and adaptive immunity. They support and maintain tissue homeostasis by promoting immune tolerance, contributing to anti-microbial defense and being essential players in tissue repair and wound healing [2,5,6,7,8]. However, monocytes are often considered double-edged swords as they also contribute to the pathogenesis and progression of chronic inflammatory conditions like inflammatory bowel disease, rheumatoid arthritis, or multiple sclerosis [9,10,11,12,13].

Over time, our understanding of monocytes has evolved from viewing them as a homogeneous macrophage precursor population to a heterogeneous population with various functions [14,15]. The first evidence for different mouse monocyte subsets was provided by Geissmann et al., who identified a CX3C motif chemokine receptor 1^high^ chemokine (C-C motif) receptor 2^−/low^ (CX3CR1^high^ CCR2^−/low^) and a CX3CR1^low^ CCR2^high^ subset with different phenotypic and functional properties, which were confirmed in later reports [15,16]. CX3CR1 is a 7-transmembrane receptor coupled to heterotrimeric G proteins that is important for the adhesion of leukocytes, cell survival, and the recruitment of immune cell subpopulations [17]. Several studies have demonstrated that CX3CR1 signaling is an essential survival factor for monocytes [18,19]. Importantly, these initial landmark studies on CX3CR1 were based on the use of reporter mouse models. In contrast, for CCR2, there was no reporter mouse model available until Saederup et al. generated *Ccr2*^RFP^ reporter mice in order to investigate monocyte subset trafficking in vivo [20]. CCR2 is a C-C chemokine receptor for the monocyte chemoattractant protein-1 (MCP-1) and has dual roles, including pro-inflammatory functions, mainly via antigen-presenting cells and T cells, and anti-inflammatory functions via regulatory T cells [21]. Furthermore, CCR2 is important for monocyte emigration from the bone marrow and efficient monocyte recruitment from the blood to inflamed tissue [22,23].

Subsequent studies further identified lymphocyte antigen 6 family member C (Ly6C) as a specific marker for discriminating monocytes in two phenotypically and functionally different subtypes [15,24]. Conventionally, driven by these studies, the literature categorizes mouse monocytes into a classical and a non-classical subset based on their inverse correlation of CX3CR1 and Ly6C/CCR2 surface expressions [1,13,25,26,27].

Classical monocytes, also called inflammatory monocytes, are defined as Ly6C^high^, CX3CR1^low^, and CCR2^high^ and are recruited to sites of inflammation at high rates, where they recognize and phagocytose pathogens and are able to attract other immune cells by secreting cytokines and anti-microbial factors. On the other hand, non-classical monocytes, defined as Ly6C^low^, CX3CR1^high^, and CCR2^−/low^, are characterized by their ability to patrol along the vascular endothelium, to remove cell debris and to promote tissue repair [27,28].

However, the phenotyping of different monocyte subsets is still ambiguous, and there are many different approaches described in the literature [28,29,30,31,32]. In the present study, we aimed to challenge the monocyte phenotyping concept based on CX3CR1, CCR2, and Ly6C by comparing surface antibody staining and genetically modified reporter mouse models.

Therefore, we used *Cx3cr1*^GFP^ and *Ccr2*^RFP^ reporter mice, in which the *Cx3cr1* or *Ccr2* gene is replaced by a *green fluorescent protein* (*GFP*) or *red fluorescent protein* (*RFP*) reporter gene, respectively. Based on this genetic background, heterozygous (*Cx3cr1*^+/GFP^ or *Ccr2*^+/RFP^) mice, with fluorescent protein substitution in only one allele, were used as reporters to identify *Cx3cr1*- or *Ccr2*-expressing monocytes, whereas homozygous mice showed a functional knock-out. While our results confirm previous work showing that there is a discrepancy between the CX3CR1 reporter gene signal and CX3CR1 surface expression, as detected by flow cytometry, we add further details to the picture by comparing the reporter signal and several validated fluorochrome-coupled antibodies head-to-head as well as different *Cx3cr1* reporter mouse lines [33,34]. Moreover, we also observed a discrepancy between the reporter and antibody signal for CCR2. However, here, we observed differential expression in flow cytometry via antibody staining, but not in reporter mice.

Taken together, our data suggest that CX3CR1 antibody surface staining, as well as *Ccr2*^RFP^ fluorescence reporter mice, should be used with caution to profile classical and non-classical monocytes.

## 2. Materials and Methods

### 2.1. Mice

All mice were used for experiments according to approval by the Animal Welfare Committee of the Government of Lower Franconia and all methods were performed according to relevant guidelines and all relevant ethical regulations. Mice were sacrificed by cervical dislocation. All animals used in this study were housed in individually ventilated cages with a regular 12 h day–night cycle and had free access to food and water at all times. *Cx3cr1^GFP^* mice (B6.129P2(Cg)-Cx3cr1^tm1Litt^/J) were received from the Jackson Laboratory and were bred in-house to C57Bl/6J mice to obtain heterozygous *Cx3cr1^+/GFP^* littermates. To receive all different haplotypes (homozygous GFP/GFP, heterozygous GFP/+, as well as wildtype +/+), heterozygous *Cx3cr1^GFP/+^* mice were mated. *Ccr2*^RFP/+^ (B6.129(Cg)-Ccr2tm2.1Ifc/J) mice were received from the Jackson Laboratory and were bred in-house, similar to the *Cx3cr1*^GFP^ strain. Age and sex-matched C57Bl/6J wildtype mice were also bred in-house. *Cx3cr1^creER^* R26-tdTomato mice (B6.Cx3cr1^tm2.1(cre/ERT2)Jung^Gt(ROSA)26Sor^tm9(CAG-tdTomato)Hze^) were available in-house. In order to induce the tdTomato reporter signal, mice were fed tamoxifen-containing food for 4 days before analysis. For all experiments, adult mice (>8 weeks) were used.

### 2.2. Isolation of Cells

Peripheral blood was collected from the facial vein. For erythrocyte removal, 2 mL of 1x BD Pharm Lyse™ lysing solution (BD Bioscience, Franklin Lakes, NJ, USA) was added to 70–80 μL of whole blood, which was vortexed and incubated for 15 min at room temperature (RT). Cells were washed two times with the FACS buffer (phosphate-buffered saline (PBS) supplemented with 1% fetal calf serum (FCS, PAN-Biotech, Aidenbach, Germany), and 2 mM EDTA) and were further processed for flow cytometry.

Splenocytes were isolated as previously described [35]. In short, freshly isolated spleens were mashed through a 40 μm cell strainer and resuspended in 3 mL of an ammonium–chloride–potassium lysis buffer (155 mM ammonium chloride; 19 mM potassium hydrogen carbonate; 0.68 mM EDTA; and pH 7.27). After 3 min, the cells were washed with PBS and counted with a Neubauer counting chamber. For flow cytometry analysis, 1–2 million splenocytes per sample were used.

### 2.3. Flow Cytometry and Fluorescence-Activated Cell Sorting (FACS)

Peripheral blood cells and splenocytes were stained for viable cells using the eBioscience Viability dye eFluor 506 or eFluor 780 (Invitrogen, Carlsbad, CA, USA) for 30 min at 4 °C and nonspecific binding was blocked using the Fc Blocking Reagent (Miltenyi Biotech, Bergisch Gladbach, Germany) according to the manufacturer’s protocol. Low-binding FACS tubes (Polypropylene round bottom tubes, FALCON, Reynosa, Tamps., Mexico) were used throughout. Cell surface staining was performed for 15 min at 4 °C using the antibodies listed in Supplemental Table S1. Fluorescently labeled cells were then fixed with 250 µL FluoroFix (BioLegend, San Diego CA, USA) for 1 h at RT, washed two times with the FACS buffer, and analyzed on an LSR Fortessa (BD Bioscience, Franklin Lakes, New Jersey, USA) instrument and with FlowJo™ v10.8 Software (BD Bioscience, Franklin Lakes, New Jersey, USA). Compensation was undertaken using single staining for each individual antibody. To investigate the expression of CX3CR1 or CCR2 on classical and non-classical monocytes, we pre-gated on Ly6C^high^ (classical) and Ly6C^low^ (non-classical) monocytes.

For FACS, peripheral blood mononuclear cells (PBMCs) were isolated from whole blood collected from the heart. Therefore, whole blood was diluted at least 1:2 in PBS and 2 mL of Lympholyte cell separation media (Cedarlane, Burlington, Ontario, Canada) was slowly layered under the cell suspension and centrifuged for 20 min at 771× *g* without break. Cells from the interphase were carefully removed and transferred into a new tube for washing. After counting, the cells were stained for flow cytometry, as mentioned above, using the antibodies listed in Supplementary Table S2. Cd11b^+^Ly6G^−^Cd115^+^Ly6C^high^ and Cd11b^+^Ly6G^−^Cd115^+^Ly6^low^ cells were sorted on an Astrios EQ Sorter (Beckman Coulter, Brea, CA, USA).

### 2.4. RNA Isolation and Quantitative Polymerase Chain Reaction (qPCR) Analysis

RNA from sorted Cd11b^+^Ly6G^−^Cd115^+^Ly6C^high^ and Cd11b^+^Ly6G^−^Cd115^+^Ly6^low^ cells was isolated using TRIzol (AMBION) according to the manufacturer’s protocol. Briefly, the cells were either directly sorted into or resuspended in 500 µL of the Trizol reagent, vortexed, and frozen at −80 °C. After thawing, 100 μL of chloroform was added, and samples were centrifuged at 20,000× *g* for 15 min at 4 °C without break to separate the protein, DNA, and RNA components. The upper aqueous phase containing the RNA was carefully collected, and 10 µg of glycogen (Thermo Fisher Scientific, Waltham, MA, USA) was added. Subsequently, 250 μL of isopropanol (Carl Roth, Karlsruhe, Germany) was added, and samples were incubated for 20 min on ice with regular vortexing intervals. Subsequently, the pellet was washed two times with 1 mL of 75% ethanol (Carl Roth, Karlsruhe, Germany) and dried at 37 °C to remove the remaining ethanol. The RNA pellet was then resuspended in 20 µL of RNase-free water and incubated for a further 10 min at 37 °C.

The concentration and purity of the extracted RNAs were measured using a Nanodrop 2000 spectrophotometer (Thermo Fisher Scientific, Waltham, MA, USA). Total RNA was transcribed into complementary DNA (cDNA) using the AffinityScript Kit (Agilent, Santa Clara, CA, USA). In short, 50 µg of mRNA were supplemented with poly d(T) primers and a random primer mix, and samples were incubated at 65 °C for 5 min at 300 rounds per minute (rpm). After incubation for 10 min at RT with reverse transcriptase, a 10x Affinity script buffer, dithiothreitol (DTT), desoxyribonucleotide triphosphate (dNTP) mixture, and RNase-free water were added. The reverse transcription was performed for 1 h at 42 °C and 300 rpm, followed by the inactivation of the enzyme at 70 °C for 15 min.

Primers for *Cx3cr1*, *enhanced-GFP* (*E-GFP*), and *HPRT* as the housekeeping gene were all purchased from Qiagen (Antwerp, Belgium). qPCR analysis was run in duplicates using SybrSelect MasterMix (Thermo Fisher Scientific, Waltham, MA, USA) and the Quantitect Primer Assay (Qiagen, Antwerp, Belgium). Duplicate values, in which the cycle threshold (Ct) value differed by more than one, were excluded from further analyses.

### 2.5. Statistics

All statistical analyses were performed using GraphPad Prism software 9.5.1. Normality was tested using the Shapiro–Wilk test. If the samples were normally distributed, a paired *t*-test was used. In contrast, if the samples were not normally distributed, a Wilcoxon test (matched pairs) was performed. Error bars in all graphs display the standard error of the mean (SEM). An α-value of *p* < 0.05 was defined as statistically significant. Significance levels are indicated by asterisks (* *p* < 0.05, ** *p* < 0.01, *** *p* < 0.001, **** *p* < 0.0001).

## 3. Results

### 3.1. Monocytes from the Peripheral Blood and Spleen of C57Bl/6 Mice Show No Inverse Correlation of Ly6C and CX3CR1 Expression by Flow Cytometry

**Figure 1:**
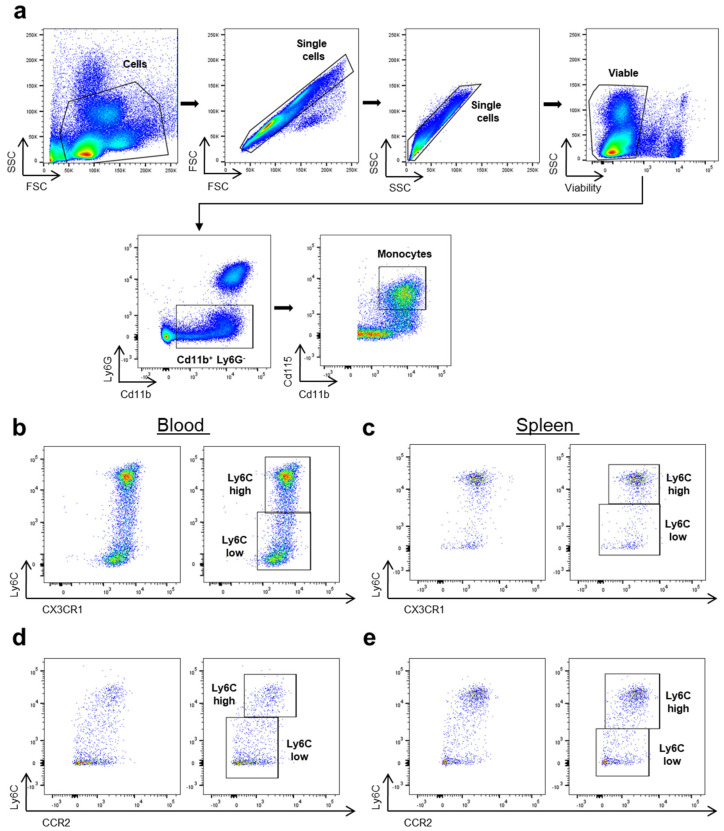
CX3CR1 antibody staining does not sufficiently discriminate mouse classical against non-classical monocytes. (**a**) Representative gating strategy to identify monocytes in C57Bl/6 (WT) peripheral blood and splenocytes. Following the exclusion of detritus based on SSC and FSC, we excluded doublets and gated on viable cells. Monocytes were further defined as Cd11b^+^Ly6G^−^ and Cd115^+^. (**b**) Ly6C (HK1.4, BioLegend) vs. CX3CR1 (QA16A03, BioLegend) expression on Cd11b^+^Ly6G^−^Cd115^+^ peripheral monocytes and representative gating for Ly6C^high^ and Ly6C^low^. (**c**) Ly6C (HK1.4, BioLegend) vs. CX3CR1 (QA16A03, BioLegend) expression on Cd11b^+^Ly6G^−^Cd115^+^ monocytes from the spleen and representative gating for Ly6C^high^ and Ly6C^low^. (**d**) Ly6C (HK1.4, BioLegend) vs. CCR2 (SA203G11, BioLegend) expression on Cd11b^+^Ly6G^−^Cd115^+^ peripheral monocytes and representative gating for Ly6C^high^ and Ly6C^low^. (**e**) Ly6C (HK1.4, BioLegend) vs. CCR2 (SA203G11, BioLegend) expression on Cd11b^+^Ly6G^−^Cd115^+^ monocytes from the spleen and representative gating for Ly6C^high^ and Ly6C^low^.

As the first step, we aimed to set up a flow cytometry panel to characterize classical and non-classical monocytes based on Ly6C and CX3CR1, as suggested in the literature [1,36,37,38]. Thus, we isolated cells from the peripheral blood of C57Bl/6 mice and analyzed them by flow cytometry. Monocytes were defined as Cd11b^+^Ly6G^−^Cd115^+^ (Figure 1a). In addition, these cells were also Cd172a^+^ and SiglecF^−^, excluding contamination with type 1 conventional DC (which are Cd172a^−^) and neutrophils (which are SiglecF^+^) (Supplemental Figure S1a). In the next step, we used Ly6C (HK1.4, BioLegend) and CX3CR1 (QA16A03, BioLegend) to subcluster these monocytes. While there was a clear separation between Ly6C^high^ and Ly6C^low^ monocytes, we did not observe any relevant differences in the CX3CR1 expression of these monocytes (Figure 1b). Similar observations were made using splenocytes from C57Bl/6 mice (Figure 1c). Furthermore, we also analyzed the correlation of Ly6C and CCR2 and, in contrast to CX3CR1, we observed the reported positive correlation between these two markers as Ly6C^high^ monocytes expressed higher levels of CCR2 (Figure 1d,e).

Collectively, these data put the inverse correlation of the surface expression of Ly6C and CX3CR1 on mouse monocytes into question.

### 3.2. Discrepancy between Fluorescent Reporter and Antibody-Based Assessment of CX3CR1 Expression

**Figure 2:**
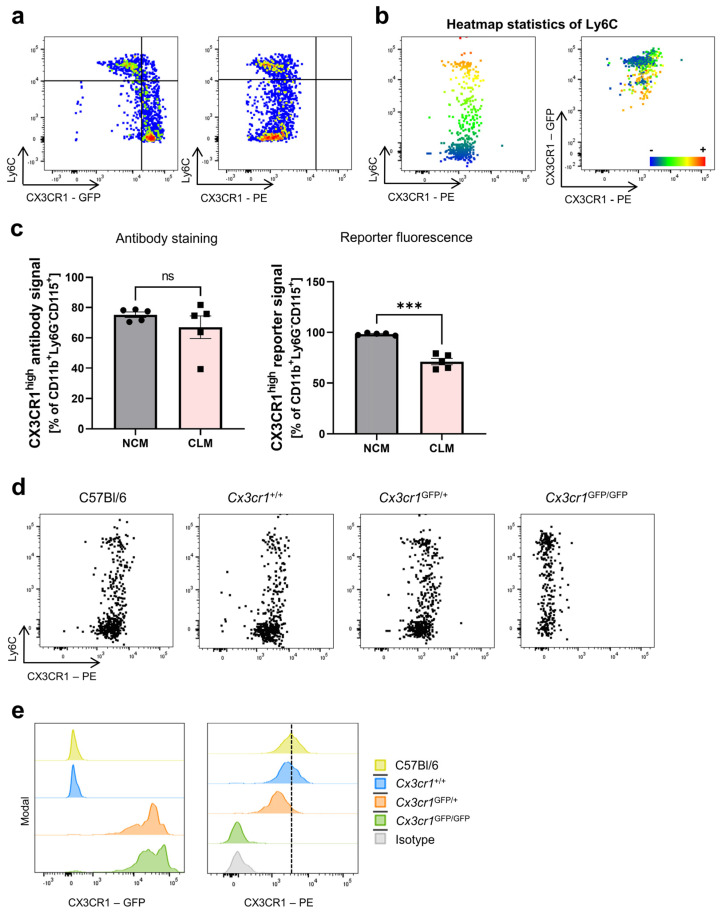
Differential CX3CR1 reporter fluorescence signal, but not antibody staining on peripheral blood monocytes in mice. (**a**) Representative flow cytometry of peripheral blood monocytes from *Cx3cr1^GFP/+^* reporter mice. Monocytes were gated as described in Figure 1. Left panel: CX3CR1-GFP fluorescence signal. Right panel: staining of the same reporter mice using the CX3CR1 (Z8-50, PE) antibody. (**b**) Dot plots visualizing the heatmap statistics of Ly6C on monocytes from *Cx3cr1^GFP/+^* reporter mice. Red indicates a high expression of Ly6C, while blue indicates a low expression of Ly6C. (**c**) Quantitative analysis of CX3CR1 expression on classical and non-classical monocytes in the peripheral blood of *Cx3cr1^GFP/+^* mice. Left panel: antibody staining (Z8-50, PE; *p* = 0.2722). Right panel: reporter fluorescence signal (*p* = 0.0005) (**d**) Representative flow cytometry showing the staining of CX3CR1 on peripheral blood monocytes using the CX3CR1 (Z8-50, PE) antibody in C57Bl/6 WT (left panel) mice, WT littermates (*Cx3cr1^+/+^*, middle left panel), heterozygous *Cx3cr1^GFP/+^* mice (right middle panel) and homozygous *Cx3cr1^GFP/GFP^* mice (right panel). (**e**) Representative histograms showing the CX3CR1-GFP and CX3CR1 (Z8-50, PE) antibody signal in C57Bl/6 (WT, yellow) mice, WT littermates (*Cx3cr1^+/+^*, blue), heterozygous *Cx3cr1^GFP/+^* (red) and homozygous *Cx3cr1^GFP/GFP^* mice (green) as well as the isotype control (grey). Data are representative of at least three independent experiments. For statistical analyses, paired *t*-tests were applied. CLM, classical monocytes; NCM, non-classical monocytes.

As the initial studies identifying classical and non-classical monocytes using CX3CR1 were based on reporter mouse models [15,39], we also investigated the surface expression of CX3CR1 and Ly6C in the peripheral blood of *Cx3cr1^GFP^* mice by flow cytometry. Therefore, we used heterozygous *Cx3cr1^GFP/+^* mice, which express *Cx3cr1* on one allele and *GFP* under the control of the *Cx3cr1* promoter on the other allele. First, we explored whether we could reproduce the inverse correlation of Ly6C and CX3CR1 expression on monocytes based on the CX3CR1 reporter signal. Indeed, we were able to distinguish a Ly6C^high^CX3CR1^GFP-low^ and a Ly6C^low^CX3CR1^GFP-high^ population in accordance with previous reports (Figure 2a) [40,41,42]. However, in line with the data shown above, we were not able to distinguish these populations on peripheral blood monocytes in the same *Cx3cr1^GFP/+^* mice (Figure 2a,b) using a CX3CR1 antibody (Z8-50, PE, BD Bioscience). Consistently, we did not observe any correlation between antibody-based CX3CR1 and Ly6C expression nor between the CX3CR1 antibody and reporter signal (Figure 2b). Furthermore, we defined classical and non-classical monocytes based on their surface expression of Ly6C and quantified the expression of CX3CR1^high^-expressing cells on both monocyte subsets. While there was no significant difference in the expression of CX3CR1 when using anti-CX3CR1 antibodies, we could clearly show a significant difference in the CX3CR1-GFP reporter fluorescence signal (Figure 2c).

Thus, in our next step, we aimed to verify whether the antibodies used were indeed functional and able to detect CX3CR1. To this end, we took advantage of different haplotypes of *Cx3cr1^GFP^* reporter mice. As expected, C57Bl/6 WT and *Cx3cr1^+/+^* mice showed no expression of CX3CR1-GFP, but substantial and similar expression of CX3CR1 was detected by antibody staining on peripheral blood monocytes. In contrast, monocytes from heterozygous *Cx3cr1^GFP/+^* mice showed moderate expression of CX3CR1-GFP as well as CX3CR1 antibody staining, which was less intense than on C57Bl/6 WT and *Cx3cr1^+/+^* monocytes. Monocytes from homozygous *Cx3cr1^GFP/GFP^* mice also expressed CX3CR1-GFP (more intense than their *Cx3cr1^GFP/+^* counterparts), but there was no CX3CR1 antibody staining as expected due to the functional knockout of CX3CR1 (Figure 2d,e). Importantly, we did not observe any differences in the abundance of monocytes in general in these different mice (Supplemental Figure S1b). Thus, together, these observations indicated that the CX3CR1 antibody used is indeed functional.

**Figure 3:**
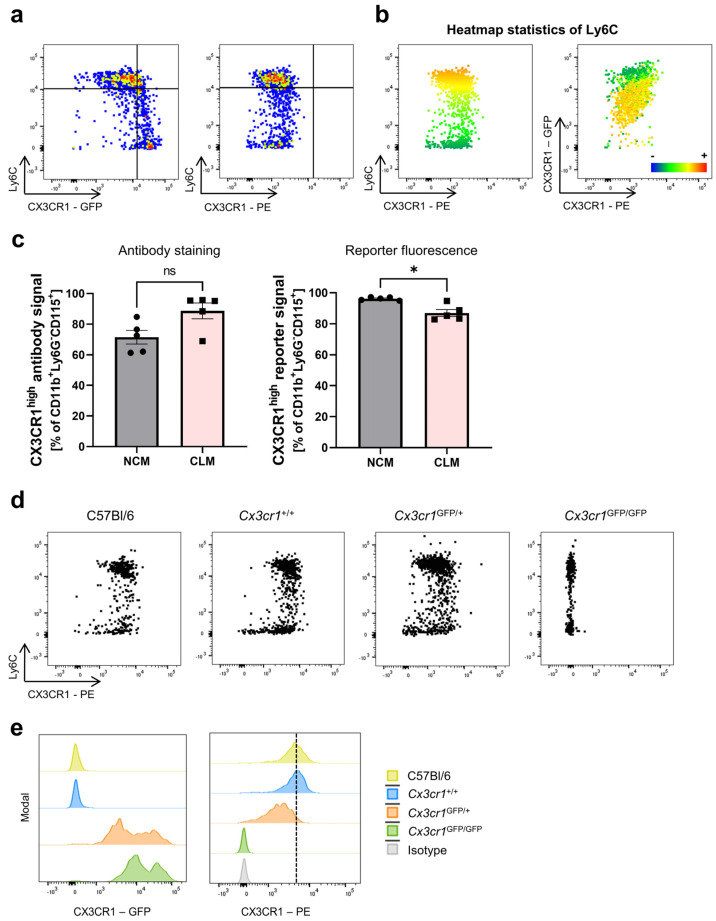
Differential CX3CR1 reporter fluorescence signal, but not antibody staining on splenic monocytes in mice. (**a**) Representative flow cytometry of GFP (left) and CX3CR1 antibody (Z8-50, PE, right) signals in monocytes from the spleen of *Cx3cr1^GFP/+^* reporter mice. (**b**) Heatmap statistics of the Ly6C surface expression on monocytes from *Cx3cr1^GFP/+^* reporter mice. Red indicates a high expression of Ly6C, while blue indicates a low expression of Ly6C. (**c**) Quantitative analysis of the CX3CR1 expression on classical and non-classical monocytes in splenocytes of *Cx3cr1*^GFP/+^ mice. Left panel: antibody staining (Z8-50, PE, *p* = 0.0625). Right panel: reporter fluorescence signal (*p* = 0.0133). (**d**) Representative flow cytometry showing CX3CR1 (Z8-50, PE) antibody staining on splenic monocytes of C57Bl/6 WT (left panel) mice, WT littermates (*Cx3cr1^+/+^*, middle left panel), heterozygous *Cx3cr1^GFP/+^* mice (right middle panel) and homozygous *Cx3cr1^GFP/GFP^* mice (right panel). (**e**) Representative histograms showing the CX3CR1-GFP and CX3CR1 (Z8-50, PE) antibody signal in C57Bl/6 (WT, yellow) mice, WT littermates (*Cx3cr1^+/+^*, blue), heterozygous *Cx3cr1^GFP/+^* mice (red) and homozygous *Cx3cr1^GFP/GFP^* mice (green) as well as the isotype control (grey). Data are representative of at least three independent experiments. For normally distributed data, the paired *t*-test was used; for not normally distributed samples, the Wilcoxon test (matched pairs) was applied. CLM, classical monocytes; NCM, non-classical monocytes.

To verify our findings across tissues, we performed the same experiments using splenocytes. As in the peripheral blood, an inverse correlation of Ly6C and CX3CR1 expression was only observed with the CX3CR1 reporter signal, but not using the CX3CR1 antibody (Z8-50, PE, BD Bioscience) (Figure 3a–c). In addition, as in the peripheral blood, we were able to prove the validity of CX3CR1 antibody staining in different *Cx3cr1* haplotypes (Figure 3d,e).

In order to exclude fluorophore- or clone-specific effects, we additionally used another four commercially available and validated CX3CR1 antibodies. However, we were not able to reproduce the inverse correlation of Ly6C and CX3CR1 expression that we observed when using the reporter signal in *Cx3cr1^GFP/+^* mice with any of them (Supplementary Figures S2 and S3).

For further validation, we also used a second *Cx3cr1* reporter mouse line, in which a tamoxifen-dependent Cre recombinase controlled by the *Cx3cr1* locus induces the expression of tdTomato (Cx3cr1^creER^ R26-tdTomato). Again, in the spleen of these mice, we observed Ly6C^high^CX3CR1^tdTomato-low^ and Ly6C^low^CX3CR1^tdTomato-high^ monocyte populations only with the CX3CR1 reporter signal, but not with antibody staining, fully supporting our findings in the *Cx3cr1^GFP^* reporter mouse model (Supplemental Figure S4).

Taken together, these data show that there is a discrepancy between CX3CR1 expression as determined by surface staining with validated CX3CR1 antibodies and the quantification of the fluorescent reporter signal in non-classical monocytes in mice.

### 3.3. mRNA Levels of Cx3cr1 and GFP Are Increased in Ly6C^low^ Compared to Ly6C^high^ Monocytes

**Figure 4:**
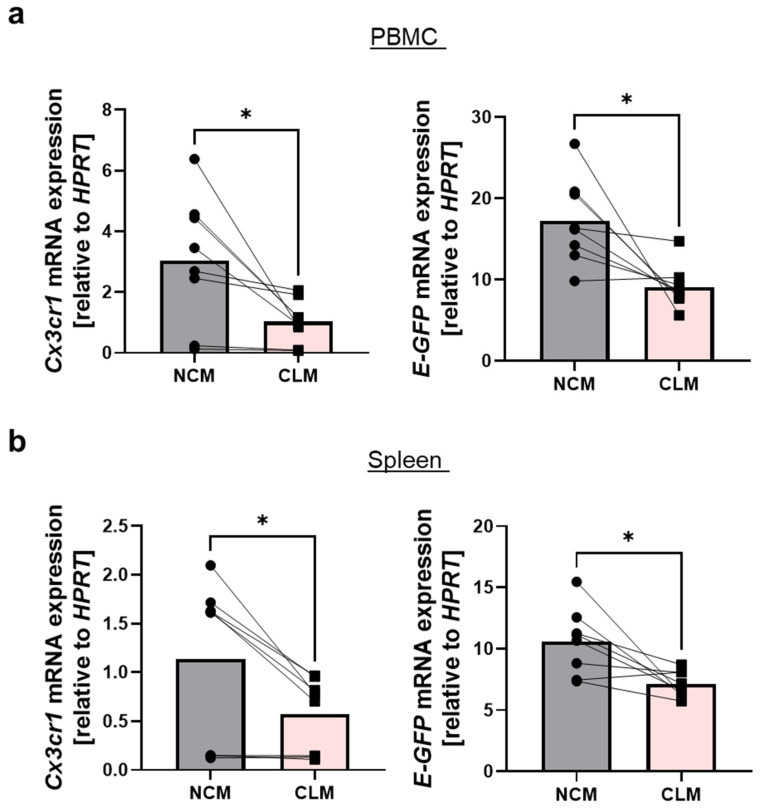
*Cx3cr1* and *GFP* expression on mRNA level in monocytes from *Cx3cr1^GFP/+^* mice. (**a**) Quantitative qPCR of *Cx3cr1* mRNA (left panel, *p* = 0.024) and *enhanced-GFP* (*E-GFP*) mRNA (right panel, *p* = 0.0144) expression relative to *HPRT* in sorted classical (Cd11b^+^Ly6G^−^Cd115^+^Ly6C^high^) and non-classical monocytes (Cd11b^+^Ly6G^−^Cd115^+^Ly6C^low^) from the peripheral blood (*n* = 8). (**b**) Quantitative qPCR of *Cx3cr1* mRNA (left panel, *p* = 0.015) and *enhanced-GFP* (*E-GFP*) mRNA (right panel, *p* = 0.0167) expression in classical and non-classical monocytes from the spleen (*n* = 8). For statistical analyses, a paired *t*-test was applied. CLM, classical monocytes; NCM, non-classical monocytes.

Finally, to better understand the reason underlying this discrepancy, we explored the expression of *Cx3cr1* and *GFP* mRNA in monocytes from *Cx3cr1^GFP/+^* mice. Therefore, we sorted Cd11b^+^Ly6G^−^Cd115^+^Ly6C^high^ classical monocytes and Cd11b^+^Ly6G^−^Cd115^+^Ly6C^low^ non-classical monocytes from the spleen and peripheral blood and performed qPCR analyses. The mRNA levels of *Cx3cr1*, as well as of *GFP*, were substantially higher in non-classical compared to classical monocytes in the blood and spleen (Figure 4). In conclusion, these data suggest that differential CX3CR1 expression in non-classical and classical monocytes in mice is lost on the surface of these cells.

### 3.4. Discrepancy between Fluorescent Reporter and Antibody-Based Assessment of Monocyte CCR2 Expression

**Figure 5:**
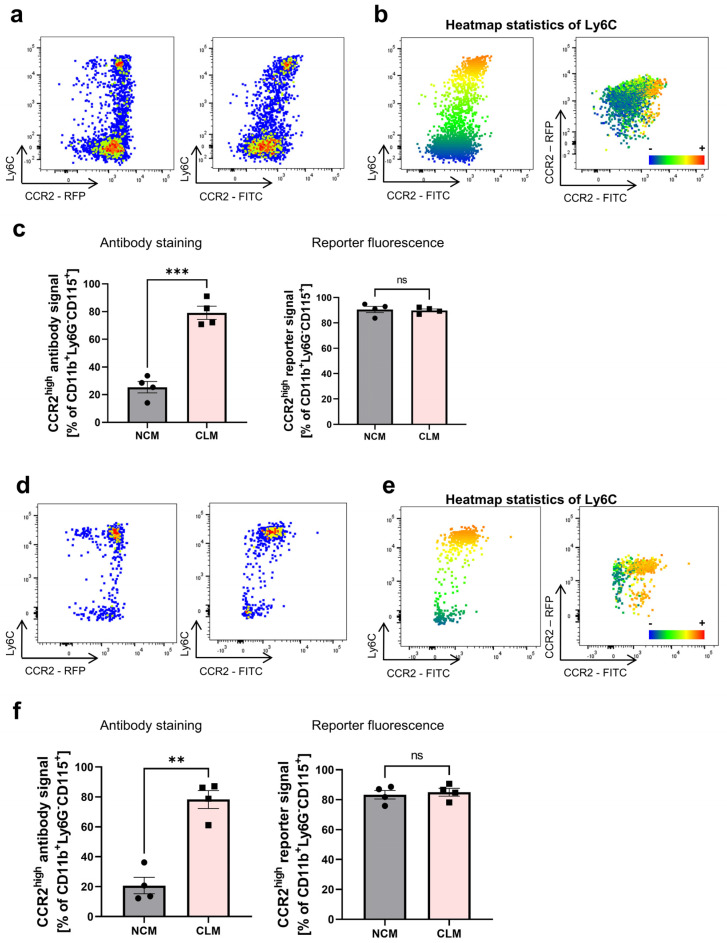
Differential CCR2 antibody staining intensity, but not reporter fluorescence signal on peripheral blood and splenic monocytes in mice. (**a**) Representative flow cytometry of peripheral blood monocytes from *Ccr2^RFP/+^* reporter mice. Monocytes were gated as described in Figure 1. Left panel: CCR2-RFP fluorescence signal. Right panel: staining of the same reporter mice using the CCR2 (SA203G11, FITC) antibody. (**b**) Dot plots visualizing the heatmap statistics of Ly6C on monocytes from *Ccr2^RFP/+^* reporter mice. Red indicates a high expression of Ly6C, while blue indicates a low expression of Ly6C. (**c**) Quantitative analysis of the CCR2 expression on Ly6C^high^ classical and Ly6C^low^ non-classical monocytes in the peripheral blood of *Ccr2^RFP/+^* mice. Left panel: antibody staining (SA203G11, FITC; *p* = 0.0008). Right panel: reporter fluorescence signal (*p* = 0.8139). (**d**) Representative flow cytometry of monocytes from the spleen of *Ccr2^RFP/+^* reporter mice. Monocytes were gated as described in Figure 1. Left panel: CCR2-RFP fluorescence signal. Right panel: staining of the same reporter mice using the CCR2 (SA203G11, FITC) antibody. (**e**) Dot plots visualizing the heatmap statistics of Ly6C on monocytes from *Ccr2^RFP/+^* reporter mice. Red indicates a high expression of Ly6C, while blue indicates a low expression of Ly6C. (**f**) Quantitative analysis of the CCR2 expression on Ly6C^high^ classical and Ly6C^low^ non-classical monocytes in the spleen of *Ccr2^RFP/+^* mice. Left panel: antibody staining (SA203G11, FITC; *p* = 0.0017). Right panel: reporter fluorescence signal (*p* = 0.5635). Data are representative of at least three independent experiments. For statistical analyses, a paired *t*-test was applied. CLM, classical monocytes; NCM, non-classical monocytes.

As CCR2 is another commonly used marker to distinguish monocyte subsets, we wanted to investigate whether there are similar differences between antibody-based and fluorescence reporter signals [15,20]. Therefore, we used heterozygous *Ccr2*^RFP^ mice, in which the coding sequence of *Ccr2* on one allele is replaced by a monomeric *RFP* sequence. We first investigated the correlation of Ly6C and CCR2 expression on Cd11b^+^Ly6G^−^Cd115^+^ monocytes in the peripheral blood. Interestingly, we did not observe the differential expression of RFP in Ly6C^high^ and Ly6C^low^ monocytes (Figure 5a). However, using a CCR2 antibody (SA203G11, FITC, BioLegend), we were able to distinguish a Ly6C^high^CCR2^high^ and a Ly6C^low^CCR2^−/low^ population in accordance with previous reports (Figure 5a,b) [43]. Consistently, we did not observe any correlation between the CCR2 antibody and the reporter signal (Figure 5b). In an additional approach, we defined classical and non-classical monocytes based on their surface expression of Ly6C and quantified the abundance of CCR2^high^ cells on both monocyte subsets using antibody staining and reporter fluorescence. While there was a strong and significant difference in the CCR2 antibody signal, we could detect no differences in the CCR2-RFP reporter fluorescence signal (Figure 5c).

In the next step, to verify our results across different tissues, we also performed these experiments with splenocytes and observed similar results. While we could not see any differences in the CCR2-RFP expression in *Ccr2*^RFP/+^ mice, there was a slight difference in the signal obtained by CCR2 antibody staining (Figure 5d,e). Consistently, when we defined the classical and non-classical monocyte subsets by their Ly6C expression and quantified the abundance of CCR2^high^ cells, we observed a significant difference in antibody staining but not in the reporter fluorescence signal (Figure 5f).

In addition, we also used a second commercially available and validated anti-CCR2 antibody to exclude fluorophore- or clone-specific effects. However, in line with our previous observations, the difference in CCR2 expression was more pronounced, when using the anti-mouse CCR2 antibody (QA18A56, PE/Cy7, BioLegend) compared to the CCR2 reporter fluorescence signal (Supplemental Figure S4c,d).

### 3.5. Differential Segregation of Classical and Non-Classical Monocytes with CX3CR1 and CCR2 Reporter Fluorescence and Antibody Staining

**Figure 6:**
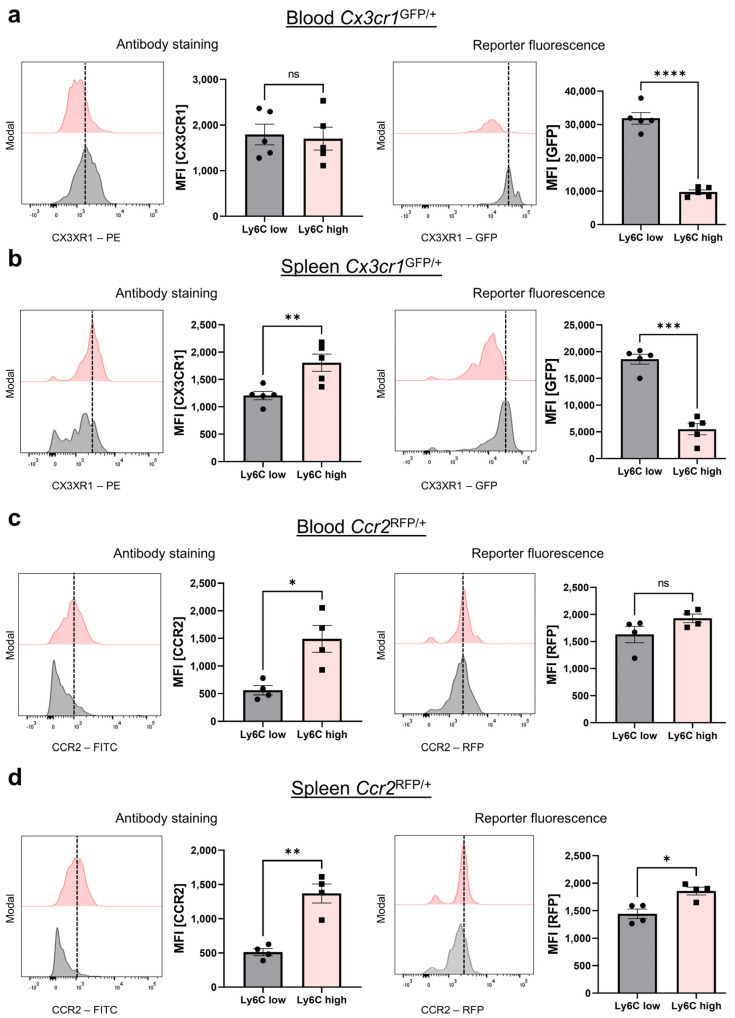
Reverse differences in the CX3CR1 and CCR2 MFI (geometric mean) in Ly6C^low^ (classical) and Ly6C^high^ (non-classical) monocytes between antibody staining and reporter fluorescence in *Cx3cr1*^GFP^ and *Ccr2*^RFP^ mice. (**a**) Representative flow cytometry histograms of peripheral blood monocytes from *Cx3cr1^GFP/+^* reporter mice and quantitative analysis of the MFI of CX3CR1 antibody staining (left panel, *p* = 0.5427) and the CX3CR1-GFP signal (right panel, *p* < 0.0001). (**b**) Representative flow cytometry histograms of monocytes from the spleen of *Cx3cr1^GFP/+^* reporter mice and quantitative analysis of the MFI of CX3CR1 antibody staining (left panel, *p* = 0.0091) and the CX3CR1-GFP signal (right panel, *p* = 0.0005). (**c**) Representative flow cytometry histograms of peripheral blood monocytes from *Ccr2^RFP/+^* reporter mice and quantitative analysis of the MFI of CCR2 antibody staining (left panel, *p* = 0.0133) and CCR2-RFP signal (right panel, *p* = 0.2470). (**d**) Representative flow cytometry histograms of splenic monocytes from *Ccr2^RFP/+^* reporter mice and quantitative analysis of the MFI of CCR2 antibody staining (left panel, *p* = 0.0053) and CCR2-RFP signal (right panel, *p* = 0.0273). Data are representative of at least three independent experiments. For statistical analyses, a paired *t*-test was applied.

Finally, we furthermore investigated the mean fluorescence intensity (MFI, geometric mean) indicative of CX3CR1 and CCR2 expression in heterozygous mice using antibody staining and the reporter signal. Once more, classical and non-classical monocytes were defined by their Ly6C expression. As expected, in the peripheral blood of *Cx3cr1*^GFP/+^ mice, we could not detect a difference in the MFI, when stained with the anti-CX3CR1 antibody (Z8-50, PE, BD Bioscience), but a strong difference, when looking at the CX3CR1-GFP reporter fluorescence (Figure 6a). Interestingly, in the spleen, we even observed a positive correlation between Ly6C expression and CX3CR1 antibody staining (Z8-50, PE, BD Bioscience) as Ly6C^high^ classical monocytes showed a higher MFI of CX3CR1 (Figure 6b). In contrast, in *Ccr2*^RFP/+^ mice, the MFI was significantly different, when CCR2 was stained with an antibody but not when the reporter fluorescence signal was assessed (Figure 6c). In the spleen, however, we observed a difference in the MFI both with antibody staining as well as for the reporter fluorescence, but this was clearly more pronounced in antibody staining (Figure 6d).

Taken together, these observations indicate that there are opposed differences in the detection of CX3CR1 and CCR2 expression by antibody staining compared to their reporter gene signal in genetically modified mouse models.

## 4. Discussion

As monocytes and downstream macrophages and DCs are essential players of the immune system and are involved in different diseases, they are potential candidates for promising therapeutic approaches [44,45,46].

In rheumatoid arthritis, classical monocytes were shown to counteract arthritis, and in contrast, the deletion of non-classical monocytes was shown to prevent mice from developing arthritis [47]. Furthermore, Butovsky et al. showed in a mouse model of Amythotrophic lateral sclerosis (ALS) that the recruitment of inflammatory monocytes to the spinal cord plays an important role in disease progression [40]. Several therapeutics are already known to affect monocytes, but therapeutic approaches or interventions that specifically target monocytes are not available. Infliximab, a chimeric anti-tumor necrosis factor (TNF) antibody, was shown to induce monocyte apoptosis, which could explain its powerful properties in patients with chronically active Crohn’s disease [48]. Hence, the accurate phenotyping of monocyte subsets is essential to derive meaningful conclusions from experimental models as well as to translate these insights into human disease.

In this study, we challenged the use of reporter mice and antibody staining in the current standard definition of mouse classical and non-classical monocytes as Ly6C^high^CX3CR1^low^CCR2^high^ and Ly6C^low^CX3CR1^high^CCR2^−/low^, respectively [27]. Indeed, based on antibody staining, we show that CX3CR1 is not differently expressed on the cell surface of classical and non-classical monocytes in mouse peripheral blood and spleens, while CCR2 is differently expressed. These observations can be explained by the fact that the initial landmark studies identifying different monocyte subsets used reporter mouse models and not antibody-based surface staining to assess CX3CR1 expression, while this was not the case for CCR2 [15]. There are several genetically modified mice that have been used to investigate monocyte migration and trafficking, including the knock-in/knock-out *Cx3cr1^GFP^* and later the *Ccr2*^RFP^ reporter strain, in which the respective genes are replaced by fluorescence reporter genes [15,20,39]. These mice have been used in many studies and differential surface expression of CX3CR1 has been assumed based on the differences in GFP expression [15,49,50,51]. However, using five different anti-CX3CR1 antibodies and two different reporter mouse models, we show that this is actually not the case, and the CX3CR1 reporter signals do not match the actual surface expression of CX3CR1, as detected by anti-CX3CR1 antibodies. These findings are consistent with a previous report by Meghraoui-Kheddar et al. They also observed that the GFP reporter signal in *Cx3cr1^GFP/+^* mice did not reflect CX3CR1 expression, as determined by a specific CX3CR1 antibody (clone SA011F11) or fluorescently labeled CX3CL1 chemokines, which are the only ligand for CX3CR1. Meghraoui-Kheddar et al. further described that Ly6C^high^ classical monocytes expressed slightly higher levels of CX3CR1 at the membrane surface and took up more soluble CX3CL1 than Ly6C^low^ non-classical monocytes in the blood, while we only observed higher CX3CR1 expression based on the MFI of classical monocytes in the spleen. Our findings indicate that CX3CR1 expression on classical and non-classical monocytes is similar in the blood [33]. In general, our approaches to analyzing the frequency of CX3CR1^high^ and CCR2^high^ expressing cells and comparing the CX3CR1 and CCR2 MFI (geometric mean) led to consistent results.

Interestingly, our data further show that on the mRNA level, *Cx3cr1* expression was higher in non-classical compared to classical monocytes. Thus, it seems that CX3CR1 reporter mouse models correctly capture differential *Cx3cr1* expression on the transcription level, while there is probably different processing for GFP and CX3CR1 at the translation or post-translational level, which maintains differences for GFP expression that are lost in the case of surface CX3CR1 detection.

Additional studies are needed to uncover the precise underlying mechanism and to determine whether, e.g., mRNA stability [52], post-translational mechanisms [53], or the transport and incorporation of the protein at the cell surface are altered [54,55].

In general, it is unknown to what extent the transcript levels by themselves dictate/predict cellular protein levels [56,57,58], and there are several examples of discrepant expression [59,60,61,62,63]. Taquet et al. showed a significant increase in somatostatin receptor 5 (SSR5) mRNA expression in Crohn’s disease patients. However, there was no increase in protein expression detected by immunohistochemistry and flow cytometry [64]. In the synovial fibroblasts from patients with rheumatoid arthritis, masparin, a proteinase inhibitor with tumor suppressive functions, was intensively expressed at the mRNA level but only slightly at the protein level [65].

It is well known that the stability of mRNAs depends on their nucleotide sequence, affecting the secondary and tertiary structure of the mRNAs and, thus, the accessibility of various proteins to bind [52]. Furthermore, several mechanisms are known to have an impact on the expression level of a protein, including translation rates, translation rate modulation, the modulation of a protein’s half-life, protein synthesis delay, and protein transport [56].

We also questioned whether there are differences between the fluorescence signal and antibody staining for the chemokine receptor CCR2. Interestingly, in contrast to the *Cx3cr1*^GFP^ reporter mouse model, we observed a different expression of CCR2 when analyzing the fluorochrome-coupled antibody but not the reporter signal. Due to the fact that CCR2 is essential for monocyte mobilization, we could not take advantage of the different genetic haplotypes, as *Ccr2*^RFP/RFP^ mice have an impaired monocyte abundance [22,23,66]. Based on our observations, we conclude that *Ccr2*^RFP^ reporter mice should be cautiously used when investigating different monocyte subsets. Further studies are warranted to explore whether this is also true for other reporter mice like the *Ccr2*^GFP^ mice designed by Satpathy and colleagues in 2010 [67].

In recent decades, alternative classification markers for monocytes have been intensely investigated. Monocytes defined by their Ly6C expression were shown to present a clear heterogeneity with regard to the expression of Cd11c, Cd43, and Cd62L, with the latter being present primarily on Ly6C^high^ monocytes, whereas Cd11c and Cd43 were found in particular on Ly6C^low^ cells [24]. Other markers that have been identified to better distinguish monocytes from other myeloid cells as well as monocyte subsets include Nr4a1, Treml4, Cd64, and Mertk [68,69,70,71,72]. Given the results of this study, it would be interesting to further investigate different technical approaches to assess their expression.

## 5. Conclusions

Beyond open questions regarding the mechanism underlying discrepant monocyte phenotyping based on the CX3CR1 and CCR2 reporter and antibody signal, the consequence of our findings is very clear: While we confirm that CX3CR1 reporter mouse models are a valuable tool to identify classical and non-classical monocytes, our data further substantiate the notion that CX3CR1 antibody-based surface staining should not be used for this purpose [33]. The discrepancy of flow cytometry gating based on reporter signal and antibody staining, as well as the discrepancy between mRNA and protein levels, should be carefully considered when investigating monocyte subsets and their functional role based on CX3CR1. In contrast, anti-CCR2 surface staining is a powerful resource to distinguish classical and non-classical monocytes and should be preferred over *Ccr2*^RFP^ reporter mice.

## Data Availability

The datasets generated and analyzed during the current study are available from the corresponding author on reasonable request. All data generated or analyzed during this study are included in this published article (and its Supplemental Information files).

## Supplementary Materials

The following supporting information can be downloaded at: https://www.mdpi.com/article/10.3390/cells13100819/s1, Figure S1: Validation of the flow cytometry gating; Figure S2: Differential CX3CR1 reporter fluorescence signal, but not antibody staining on peripheral blood monocytes in mice using different validated anti-CX3CR1 antibodies; Figure S3: Differential CX3CR1 reporter fluorescence signal, but not antibody staining on splenic monocytes in mice using different validated anti-CX3CR1 antibodies; Figure S4: Validation of the differential CX3CR1 reporter gene signal and antibody staining in tamoxifen-inducible Cx3cr1 reporter mice and *Ccr2*^RFP^ reporter mice using a second validated fluophore-coupled antibody; Table S1: List of antibodies used for flow cytometry; Table S2: List of antibodies used for fluorescence-activated cell sorting.
