# Supplementary material for: Discrepant Phenotyping of Monocytes Based on CX3CR1 and CCR2 Using Fluorescent Reporters and Antibodies
Source: Cells. 2024 May 10;13(10):819. doi: 10.3390/cells13100819 (PMC11119841; doi:10.3390/cells13100819)
Supplement: Supplementary file 1 [file cells-13-00819-s001.zip › cells-2975240-supplementary-proofed.pdf]

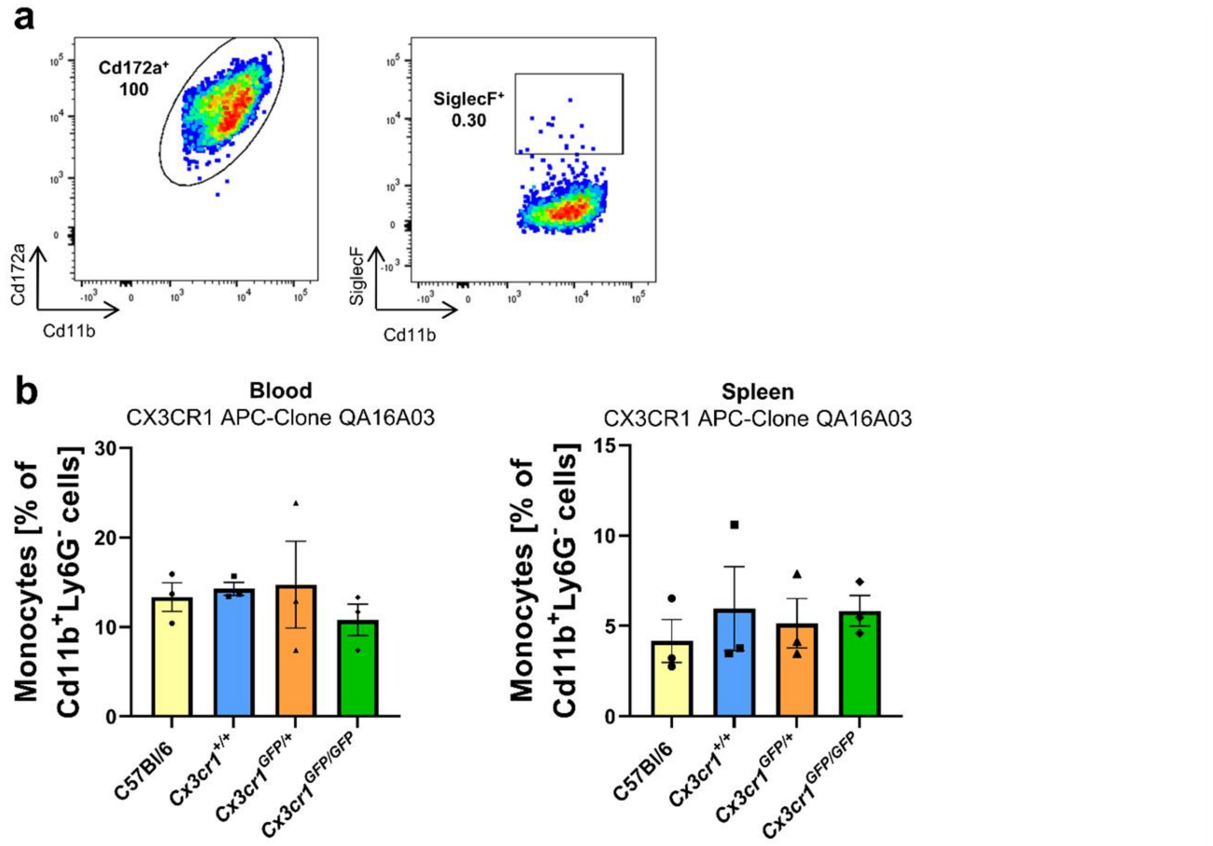

__Supplemental Figure S1:__ Validation of the flow cytometry gating **(a)** Expression of Cd172a and SiglecF on monocytes as identified in *Figure 1*. **(b)** Quantification of $Cd11b^{+}Ly6G^{-}Cd115^{+}$ monocytes in C57Bl/6 WT mice, WT littermates (*Cx3cr1*$^{+/+}$), heterozygous *Cx3cr1*$^{GFP/+}$ and homozygous *Cx3cr1*$^{GFP/GFP}$ mice in the peripheral blood (left panel) and the spleen (right panel; $n = 3$). Similar results were obtained with all other CX3CR1 antibody clones used in this study.

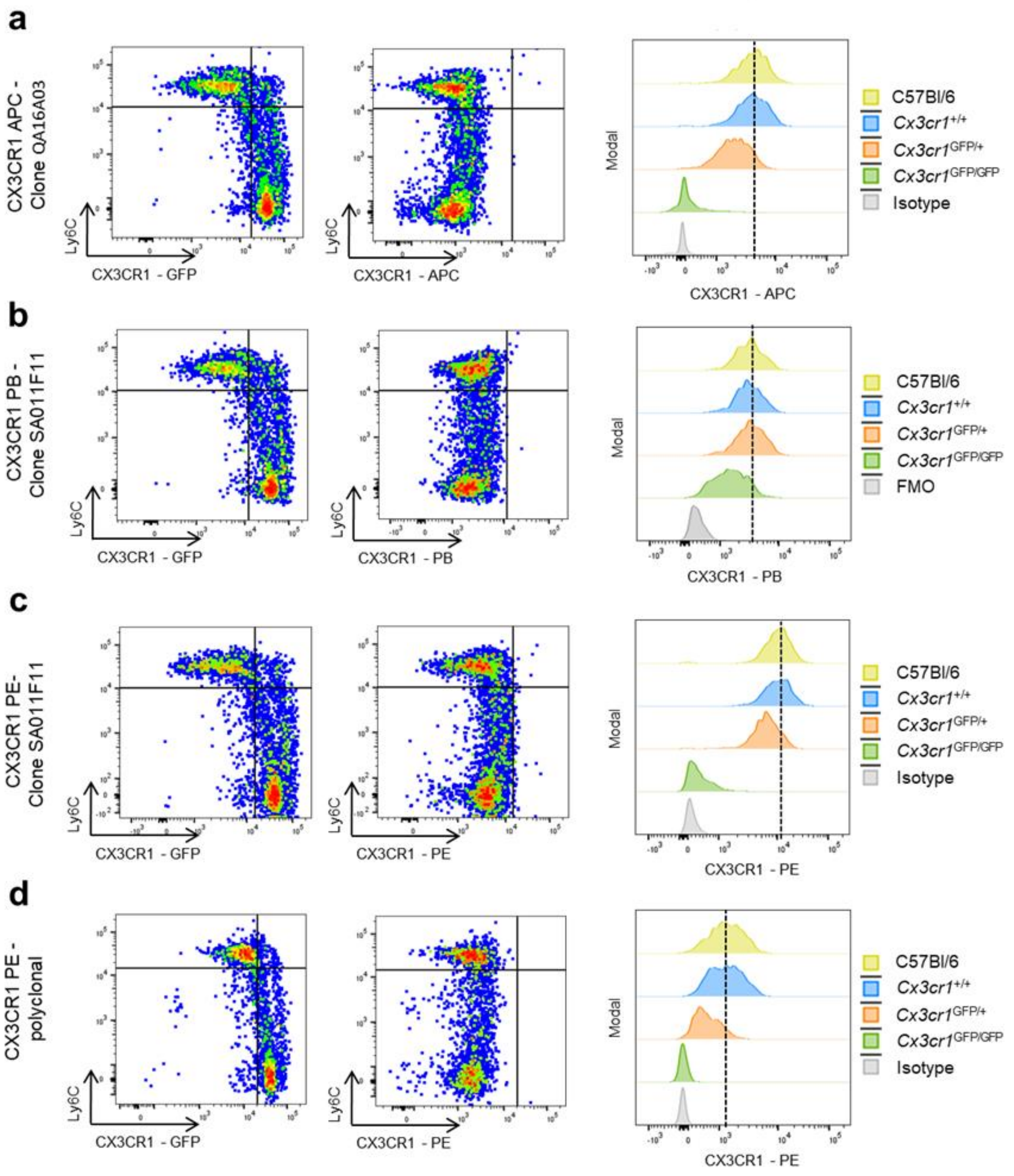

Supplemental Figure S2: Differential CX3CR1 reporter fluorescence signal, but not antibody staining on peripheral blood monocytes in mice using different validated anti-CX3CR1 antibodies. **(a-d)** Representative flow cytometry showing the GFP signal (left panels) or antibody staining of CX3CR1 clone QA16A03 (APC, BioLegend) **(a)**, CX3CR1 clone SA011F11 (PB, BioLegend) **(b)**, CX3CR1 clone SA011F11 (PE, BioLegend) **(c)** or CX3CR1 polyclonal (PE, R&D) **(d)** (middle panel) of peripheral blood monocytes from the same *Cx3cr1*$^{GFP/+}$ reporter mice. Right panels: representative histograms of CX3CR1 antibody staining on monocytes from C57Bl/6 (WT, yellow) mice, WT littermates (*Cx3cr1*$^{+/+}$, blue), heterozygous *Cx3cr1*$^{GFP/+}$ mice (red), homozygous *Cx3cr1*$^{GFP/GFP}$ mice (green) and isotype or fluorescence minus one (FMO) control (grey).

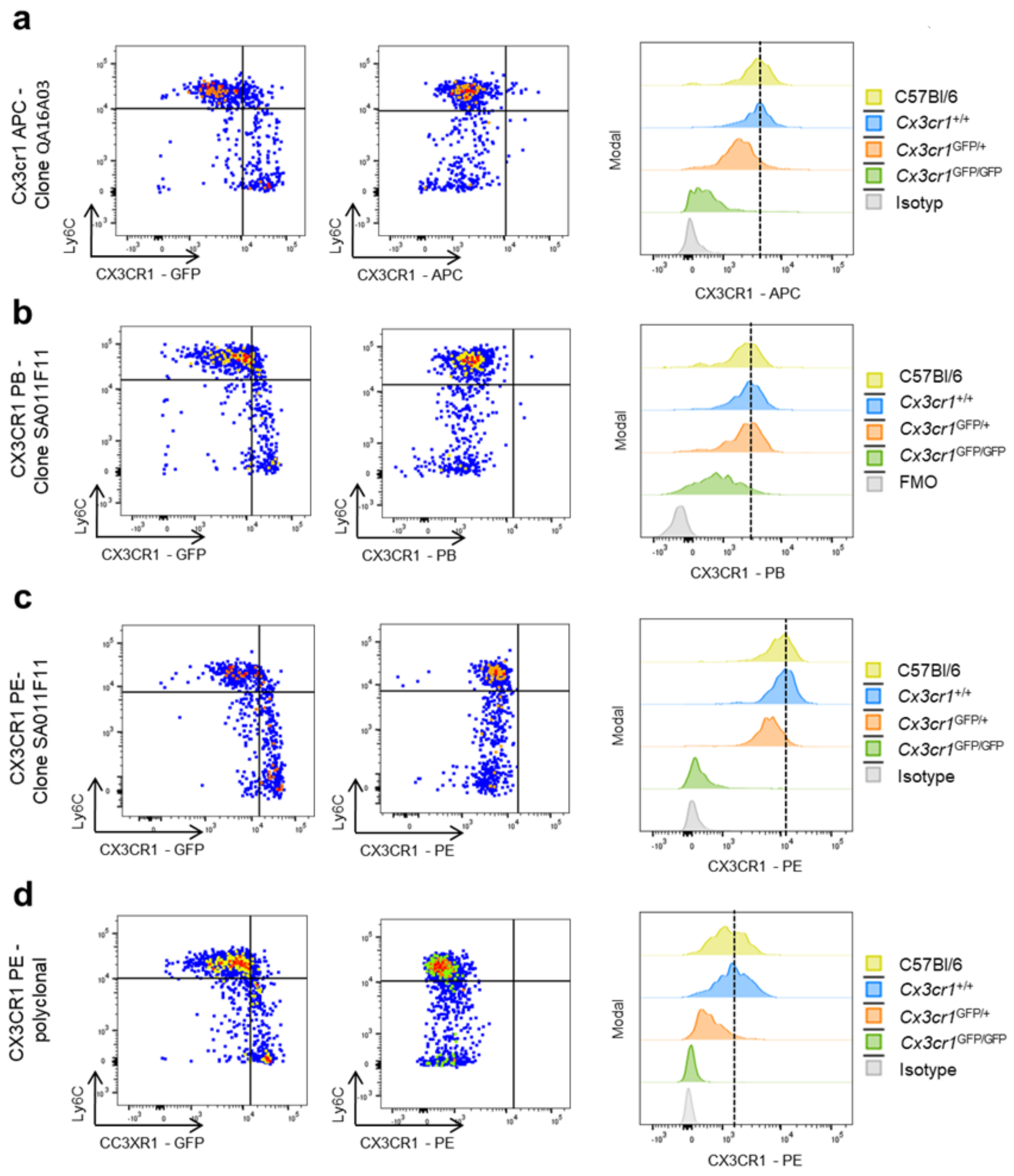

__Supplemental Figure S3:__ Differential CX3CR1 reporter fluorescence signal, but not antibody staining on splenic monocytes in mice using different validated anti-CX3CR1 antibodies. **(a-d)** Representative flow cytometry showing the respective GFP signal (left panels) or antibody staining of CX3CR1 clone QA16A03 (APC, BioLegend) **(a)**, CX3CR1 clone SA011F11 (PB, BioLegend) **(b)**, CX3CR1 clone SA011F11 (PE, BioLegend) **(c)** and CX3CR1 polyclonal (PE, R&D) **(d)** of splenic monocytes from the same heterozygous *Cx3cr1*$^{GFP/+}$ reporter mice. Right panels: representative histograms of antibody staining in monocytes from the spleen of C57Bl/6 (WT, yellow) mice, WT littermates (*Cx3cr1*$^{+/+}$, blue), heterozygous *Cx3cr1*$^{GFP/+}$ mice (red), homozygous *Cx3cr1*$^{GFP/GFP}$ mice (green) and isotype or fluorescence minus one (FMO) control (grey), respectively.

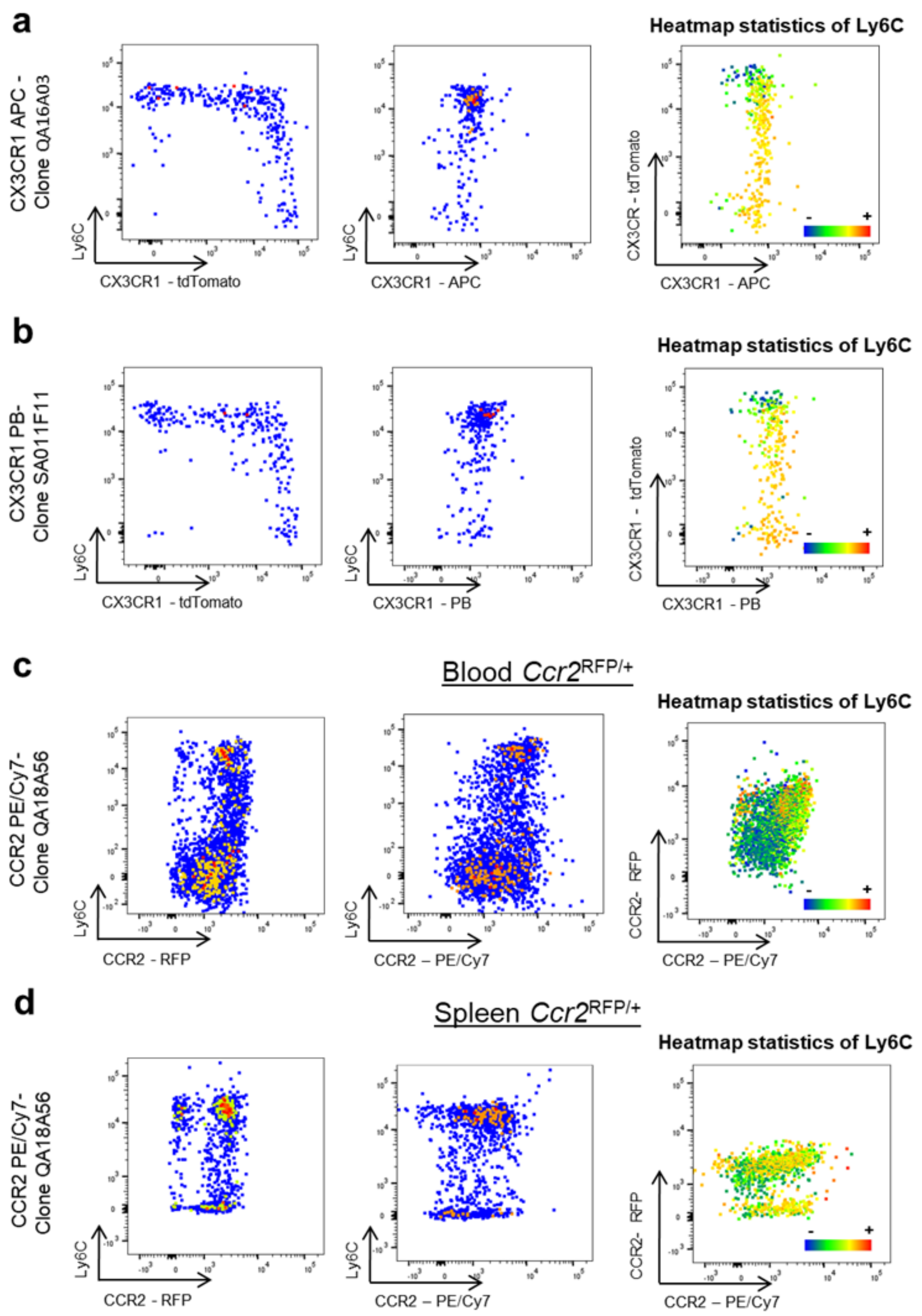

Supplemental Figure S4: **(a, b)** Validation of the differentially CX3CR1 reporter gene signal and antibody staining in tamoxifen-inducible *Cx3cr1* reporter mice. **(a)** Left panels: representative flow cytometry showing the CX3CR1-tdTomato signal or CX3CR1 (QA16A03, APC) antibody signal in monocytes from the spleen of tamoxifen inducible *Cx3cr1* reporter mice. Right panel: visualisation of the heatmap statistics of Ly6C expression. Red indicates high expression of Ly6C, while blue indicates low expression of Ly6C.

(b) Left panels: representative flow cytometry showing the CX3CR1-tdTomato signal or CX3CR1 (SA011F11, PacificBlue) antibody staining in monocytes from the spleen of tamoxifen-inducible *Cx3cr1* reporter mice. Right panel: heatmap statistics of Ly6C expression.

(c, d) Differential CCR2 antibody staining, but not reporter fluorescence signal on **(c)** peripheral blood monocytes and **(d)** splenic monocytes. Representative flow cytometry showing the RFP signal (left panels) or antibody staining of CCR2 clone QA18A56 (PE/Cy7, BioLegend) from the same *Ccr2*$^{RFP/+}$ reporter mice. Right panels: representative dot plots visualizing the heatmap statistics of Ly6C on monocytes from *Ccr2*$^{RFP/+}$ reporter mice. Red indicates a high expression of Ly6C while blue indicates low expression of Ly6C.

Supplemental Table S1: List of antibodies used for flow cytometry

Target	Fluorophore	Clone	Manufacturer	Dilution factor
Cd11b	BV711	M1/80	BioLegend	1:100
Ly6G	PerCp/Cy5.5	1A8	BioLegend	1:100
Cd115	BV605	AF598	BioLegend	1:50
Ly6C	APC/Cy7	HK1.4	BioLegend	1:100
Ly6C	FITC	HK1.4	BioLegend	1:200
SiglecF	BV421	E50-2440	BD Bioscience	1:200
Cd172a	PE/Cy7	P84	BioLegend	1:80
CX3CR1	APC	QA16A03	BioLegend	1:100
CX3CR1	Pacific Blue	SA011F11	BioLegend	1:200
CX3CR1	PE	SA011F11	BioLegend	1:600
CX3CR1	PE	Z8-50 (Z8-50.23)	BD Bioscience	1:200
CX3CR1	PE	Polyclonal Goat IgG	R&D Systems	1:10
Mouse IgG1, k	APC	MOPC-21	BioLegend	1:100
Mouse IgG2a, k	PE	MOPC-173	BioLegend	1:600
Rat IgG2a, k	PE	RTK2758	BioLegend	1:200
Goat IgG	PE	Polyclonal	R&D Systems	1:10
CCR2	PE/Cy7	QA18A56	BioLegend	1:50
CCR2	FITC	SA203G11	BioLegend	1:50
Rat IgG2b, k	PE/Cy7	RTK4530	BioLegend	1:50
Rat IgG2b, k	FITC	RTK4530	BioLegend	1:50

Supplemental Table S2: List of antibodies used for fluorescence-activated cell sorting

Target	Fluorophore	Clone	Manufacturer	Dilution factor
Cd11b	APC	M1/80	BioLegend	1:100
Ly6G	BV421	1A8	BioLegend	1:100
Cd115	PE/Cy7	AFS98	BioLegend	1:50
Ly6C	APC/Cy7	HK1.4	BioLegend	1:100
